# Association between controlling nutritional status score and left ventricular hypertrophy in adults: A single-center retrospective cross-sectional study

**DOI:** 10.1097/MD.0000000000050792

**Published:** 2026-09-25

**Authors:** Guan-Ying Yang, Jing Tang, Long Yang, Yan-Hong Li

**Affiliations:** a State Key Laboratory of Pathogenesis, Prevention, and Treatment of High Incidence Diseases in Central Asia, Xinjiang Medical University, Urumqi, China; b Pharmacy Department, Fifth Affiliated Hospital of Xinjiang Medical University, Urumqi, China; d Department of Thyroid and Breast Surgery, General Hospital of Xinjiang Military Region, Urumqi, China.; c Clinical Laboratory, First Affiliated Hospital of Xinjiang Medical University, Urumqi, China

**Keywords:** controlling nutritional status, left ventricular ejection fraction, left ventricular hypertrophy, left ventricular mass index

## Abstract

Left ventricular hypertrophy (LVH) is an important marker of cardiovascular risk, while malnutrition is common among adults. The Controlling Nutritional Status (CONUT) score is an objective tool for nutritional assessment. This study aimed to investigate the associations of CONUT-defined nutritional status with LVH and reduced left ventricular ejection fraction (LVEF) in adults. This single-center retrospective cross-sectional study included 2066 adults. Nutritional status was classified according to the standard CONUT categories as normal nutrition, mild malnutrition, and moderate/severe malnutrition. Echocardiography was used to assess left ventricular mass index and LVEF, and LVH was defined using sex-specific left ventricular mass index thresholds. Logistic regression analyses were performed to evaluate the associations of CONUT-defined nutritional status with LVH and reduced LVEF. Among the 2066 participants, 1002 (48.5%) had normal nutritional status, 912 (44.1%) had mild malnutrition, and 152 (7.4%) had moderate/severe malnutrition. In the multivariable logistic regression model, both mild malnutrition (odds ratio [OR] = 1.597, 95% confidence interval [CI]: 1.033–2.495; *P* = .037) and moderate/severe malnutrition (OR = 2.810, 95% CI: 1.445–5.223; *P* = .002) were independently associated with higher odds of LVH. Moderate/severe malnutrition was independently associated with reduced LVEF (OR = 2.350, 95% CI: 1.259–4.239; *P* = .006). Poorer CONUT-defined nutritional status was associated with a higher prevalence of left ventricular structural and functional abnormalities. CONUT-defined nutritional status may provide supplementary information for cardiovascular risk stratification.

## 1. Introduction

Cardiovascular disease (CVD) is the primary cause of mortality worldwide, with more than 80% of cases occurring in low-and middle-income countries.^[1]^ Left ventricular hypertrophy (LVH) doubles the risk of CVD events and all-cause mortality.^[2]^ LVH is a broad term. Hypertrophy is defined as the enlargement of an organ or its parts, resulting from the growth of its components, in contrast to hyperplasia, which involves an increase in the number of components. LVH refers to the enlargement of the left ventricle or its components, resulting from cardiomyocyte hypertrophy. The etiology of LVH is diverse, encompassing conditions such as hypertension, valvular diseases, cardiomyopathies, athletic adaptation, obesity, metabolic syndrome, myocardial infarction, amyloidosis, and Chagas disease.^[3]^ Left ventricular mass index (LVMI) is a crucial metric for assessing LVH. Left ventricular ejection fraction (LVEF) is widely used in clinical practice as a measure of cardiac performance, with values typically considered normal at 50%.^[4]^ Timely diagnosis and treatment have been associated with more favorable cardiac profiles in patients with LVH in prior clinical studies.^[5]^

The impact of nutritional status on various CVDs has been increasingly scrutinized because of its modifiable nature compared to other clinical factors.^[6]^ Previous research has primarily concentrated on overnutrition, reporting strong cross-sectional correlations between overnutrition and CVD markers.^[7]^ Recent cross-sectional and cohort studies have reported that malnutrition is significantly associated with poorer prognosis in patients with acute coronary artery disease, heart failure, atrial fibrillation, and valvular heart disease.^[8–10]^

Body mass index, serum albumin, and prealbumin are commonly used clinical indicators for assessing nutritional status. However, the clinical utility of individual nutritional markers is limited because they may reflect only selected dimensions of nutritional status. Researchers have developed refined nutritional indices integrating multiple laboratory indicators.^[11]^ The Controlling Nutritional Status (CONUT) score, derived from serum albumin levels, total peripheral lymphocyte count, and total cholesterol (TC) concentration, serves as a screening tool for early identification of poor nutritional status.^[12]^ Several cross-sectional and cohort studies have reported correlations between elevated CONUT scores and higher inpatient mortality rates in cancer, CVD, and internal medicine.^[9,13–18]^

Nevertheless, evidence regarding the association between CONUT-defined nutritional status and left ventricular structural abnormalities in adults remains limited. In particular, few studies have evaluated whether the severity of malnutrition according to standard CONUT categories is associated with a higher prevalence of clinically defined LVH and impaired left ventricular systolic function. Therefore, this study aimed to investigate the associations of CONUT-defined nutritional status with LVH, LVMI and LVEF in a large adult population, thereby providing observational evidence for cardiovascular risk stratification.

## 2. Methods

### 2.1. Study design and participants

This retrospective cross-sectional study included 2066 adults who attended the Cardiovascular Department Outpatient Clinic of the First Affiliated Hospital of Xinjiang Medical University between January and March 2025. The Medical Ethics Committee of the First Affiliated Hospital of Xinjiang Medical University approved the research protocol (Approval Number: K202508-12). Due to the retrospective design of this study, the requirement for informed consent was waived by the ethics committee. Inclusion criteria: Age >18 years, (2) completion of echocardiography, and (3) completion of laboratory tests, including routine blood tests and liver and kidney function assessments. Exclusion criteria: Congenital heart disease and severe heart valve diseases, including mitral stenosis, mitral regurgitation, aortic stenosis, and aortic insufficiency. Pericardial diseases include acute pericarditis and constrictive pericarditis. Heart failure caused by non-cardiogenic factors (severe infection and anemia). Abnormal thyroid function. Severe liver injury (glutathione and glutamate-oxaloacetic acid aminotransferase levels exceeded the upper limit of normal by 3 times). Severe renal insufficiency. Malignant tumors.

### 2.2. General clinical data collection and laboratory tests

All clinical data were obtained from the outpatient electronic medical record system at the initial visit. Demographic and lifestyle information, including age, sex, smoking status, and alcohol consumption, was collected from the medical records. Height, weight, systolic blood pressure, and diastolic blood pressure were measured by trained outpatient nurses using standardized procedures. Body mass index was calculated as weight in kilograms divided by height in meters squared. Clinical information regarding hypertension, diabetes, coronary heart disease (CHD), New York Heart Association functional class, and current medication use was also recorded. Medication use included statins, angiotensin-converting enzyme inhibitors or angiotensin receptor blockers (ACEI/ARBs), β-blockers, calcium channel blockers (CCBs), and diuretics. Fasting venous blood samples were collected at the initial visit for laboratory assessment. The laboratory variables included white blood cell count, neutrophil count, lymphocyte count, fasting blood glucose, triglycerides, TC, high-density lipoprotein cholesterol, low-density lipoprotein cholesterol (LDL-C),

### 2.3. Echocardiographic evaluation

Echocardiographic examinations were performed using a VIVID7 color Doppler ultrasound system (GE, USA). Standard echocardiographic parameters, including left ventricular end-diastolic diameter (LVDd), interventricular septal thickness (IVST), left ventricular posterior wall thickness (LVPWT), and LVEF, were measured according to standardized procedures. Left ventricular mass (LVM) was calculated using the Devereux formula as follows: LVM (g) = 0.8 × 1.04 × [(LVDd + IVST + LVPWT)^3^ − LVDd^3^] + 0.6. Body surface area (BSA) was calculated using the Stevenson formula developed for Chinese adults: BSA (m^2^) = 0.0061 × height (cm) + 0.0128 × weight (kg)−0.1529. LVMI was calculated as:LVMI (g/m^2^) = LVM (g)/BSA (m^2^). Considering population-specific differences in cardiac structural reference values, LVH was defined according to the sex-specific LVMI thresholds adopted in the Chinese Guidelines for the Management of Hypertension.^[19]^ LVH was defined as an LVMI >125 g/m^2^ in men and >120 g/m^2^ in women. Left ventricular systolic function was categorized as preserved when LVEF was ≥50% and reduced when LVEF was <50%.

### 2.4. Diagnosis of CHD, hypertension and diabetes

CHD was identified based on documented medical history and/or coronary angiography demonstrating ≥50% luminal stenosis in at least 1 major coronary artery. Hypertension was defined according to a documented history of hypertension, current use of antihypertensive medications, or systolic blood pressure ≥140 mm Hg and/or diastolic blood pressure ≥90 mm Hg. Diabetes mellitus was defined based on a documented history of diabetes, current use of glucose-lowering medications or insulin, glycated hemoglobin (HbA1c) ≥6.5%, fasting plasma glucose ≥7.0 mmol/L, or random plasma glucose ≥11.1 mmol/L.

### 2.5. Definition of CONUT score

The CONUT score was calculated using serum albumin concentration, TC concentration, and total peripheral lymphocyte count. Each component was assigned a predefined score according to the standard CONUT scoring system, and the component scores were summed to obtain the total CONUT score, with higher scores indicating poorer nutritional status (Table 1).^[20]^ According to the standard CONUT classification, participants were categorized as having normal nutritional status, mild malnutrition, moderate malnutrition, or severe malnutrition. Because the number of participants with severe malnutrition was relatively small, participants with moderate and severe malnutrition were combined into a single category to improve statistical stability.

**Table 1:** CONUT nutritional status scoring criteria.

Characteristics	Grade			
Albumin(g/L)	≥35	30–34.9	25–29.9	<25
score	0	2	4	6
Cholesterol(mg/dL)	>180	140–180	100–139	<100
score	0	1	2	3
Lymphocyte count(10^9^/L)	≥1.6	1.2–1.59	0.8–1.19	<0.8
score	0	1	2	3
CONUT	0–1	2–4	5–8	9–19
	Normal	Mild	Moderate	Severe

CONUT = Controlling Nutritional Status.

### 2.6. Statistical analyses

Statistical analyses were performed using R software (version 4.2.3). All statistical tests were 2-sided, and a *P* value <.05 was considered statistically significant. Continuous variables were expressed as mean ± standard deviation when normally distributed and as median (interquartile range [IQR]) when non-normally distributed. Categorical variables were presented as numbers and percentages. For comparisons among the 3 groups, normally distributed continuous variables were analyzed using 1-way analysis of variance, whereas non-normally distributed variables were analyzed using the Kruskal–Wallis test. For comparisons between 2 groups, continuous variables were analyzed using Student *t* test or the Mann–Whitney *U* test, as appropriate, and categorical variables were analyzed using the Pearson chi-square test. Univariable and multivariable logistic regression analyses were performed to evaluate the associations of CONUT-defined nutritional status and other clinical variables with LVH and reduced (LVEF < 50%). The final multivariable models were determined using bidirectional stepwise regression.

## 3. Results

### 3.1. Clinical characteristics of adults with malnutrition group versus normal nutrition group

A total of 2066 participants were included in the study, of whom 1002 (48.5%) had normal nutritional status, 912 (44.1%) had mild malnutrition, and 152 (7.4%) had moderate/severe malnutrition according to the CONUT classification. Significant differences across the 3 groups were observed in sex distribution, hypertension, diabetes, CHD, and New York Heart Association functional class (all *P* < .05). The use of statins, ACEI/ARBs, β-blockers, CCBs, and diuretics also differed significantly among the 3 groups (all *P* < .05). Regarding laboratory parameters, significant between-group differences were found in neutrophil count, lymphocyte count, estimated glomerular filtration rate (eGFR), glucose, triglycerides, TC, high-density lipoprotein cholesterol, LDL-C, total bilirubin, and albumin (all *P* < .05), whereas white blood cell count did not differ significantly. Echocardiographic parameters including LA, LVDd, LVPWT, LVMI, and LVEF differed significantly across the 3 nutritional status groups, while IVST did not. The prevalence of LVH increased from 3.6% in the normal nutrition group to 5.9% in the mild malnutrition group and 9.9% in the moderate/severe malnutrition group (*P* < .01). Similarly, the prevalence of LVEF < 50% increased from 4.6% to 7.3% and 12.5%, respectively (*P* < .001) (Table 2).

**Table 2:** Baseline characteristics according to CONUT nutritional status.

Characteristic	Total	Normal nutrition	Mild malnutrition	Moderate/severe malnutrition	P value
	(n = 2066)	(n = 1002)	(n = 912)	(n = 152)	
**General characteristics**					
Age, yr	55.0 (49.0, 63.0)	55.0 (48.0, 63.0)	55.5 (49.0, 62.0)	55.0 (50.0, 64.0)	0.561
Male, n (%)	1121 (54.3)	502 (50.1)	544 (59.6)	75 (49.3)	<0.001
BMI, kg/m^2^	26.1 (23.8, 29.0)	26.2 (24.0, 29.1)	26.0 (23.7, 28.7)	26.2 (23.5, 29.1)	0.373
Smoking, n (%)	866 (41.9)	411 (41.0)	395 (43.3)	60 (39.5)	0.488
Drinking, n (%)	357 (17.3)	160 (16.0)	175 (19.2)	22 (14.5)	0.113
SBP, mm Hg	136.0 (118.0, 167.0)	133.5 (118.0, 161.0)	138.5 (117.0, 170.0)	137.0 (120.0, 164.2)	0.297
DBP, mm Hg	82.0 (72.0, 100.0)	81.0 (72.0, 98.0)	83.0 (70.0, 100.0)	84.5 (75.8, 100.0)	0.24
Hypertension, n (%)	800 (38.7)	348 (34.7)	403 (44.2)	49 (32.2)	<0.001
Diabetes, n (%)	270 (13.1)	109 (10.9)	143 (15.7)	18 (11.8)	0.007
CHD, n (%)	1112 (53.8)	499 (49.8)	530 (58.1)	83 (54.6)	0.001
NYHA class, n (%)					0.015
I	396 (35.6)	180 (36.1)	174 (32.8)	42 (50.6)	
II	634 (57.0)	286 (57.3)	315 (59.4)	33 (39.8)	
III or IV	82 (7.4)	33 (6.6)	41 (7.7)	8 (9.6)	
**Medication use**					
Statin use, n (%)	1041 (50.4)	458 (45.7)	513 (56.2)	70 (46.1)	<0.001
ACEI/ARB use, n (%)	863 (41.8)	376 (37.5)	423 (46.4)	64 (42.1)	<0.001
β-blocker use, n (%)	984 (47.6)	425 (42.4)	485 (53.2)	74 (48.7)	<0.001
CCB use, n (%)	1075 (52.0)	496 (49.5)	509 (55.8)	70 (46.1)	<0.010
Diuretic use, n (%)	499 (24.2)	216 (21.6)	243 (26.6)	40 (26.3)	0.028
**Laboratory findings**					
WBC, 10^9^/L	6.59 (5.51, 7.82)	6.62 (5.62, 7.86)	6.50 (5.40, 7.71)	6.58 (5.33, 8.31)	0.203
Neutrophil count, 10^9^/L	3.75 (2.98, 4.66)	3.65 (2.93, 4.51)	3.81 (3.09, 4.74)	3.80 (2.97, 5.11)	0.009
Lymphocyte count, 10^9^/L	2.05 (1.66, 2.53)	2.21 (1.87, 2.67)	1.88 (1.47, 2.37)	1.83 (1.33, 2.38)	<0.001
eGFR, mL/min/1.73 m^2^	101.1 (89.3, 109.0)	102.1 (90.9, 109.5)	100.1 (88.8, 108.0)	99.9 (87.6, 108.1)	0.007
Glucose, mmol/L	5.04 (4.52, 6.04)	5.03 (4.53, 5.84)	5.02 (4.47, 6.14)	5.34 (4.59, 6.77)	0.014
Triglycerides, mmol/L	1.41 (1.01, 2.08)	1.57 (1.13, 2.29)	1.27 (0.91, 1.86)	1.36 (0.91, 2.10)	<0.001
Total cholesterol, mg/dL	153.9 (126.9, 182.0)	176.3 (158.2, 200.7)	127.4 (111.4, 144.3)	136.3 (112.1, 167.5)	<0.001
HDL-C, mmol/L	1.03 (0.86, 1.26)	1.09 (0.91, 1.31)	0.98 (0.81, 1.18)	0.97 (0.78, 1.30)	<0.001
LDL-C, mmol/L	2.53 (1.96, 3.13)	3.00 (2.61, 3.50)	2.01 (1.65, 2.42)	1.99 (1.57, 2.57)	<0.001
Albumin, g/L	40.2 (37.5, 42.8)	41.1 (38.9, 43.4)	40.0 (37.4, 42.4)	26.6 (20.6, 31.9)	<0.001
**Echocardiographic parameters**					
LA, cm	3.30 (3.10, 3.60)	3.30 (3.00, 3.60)	3.40 (3.10, 3.70)	3.50 (3.10, 4.00)	<0.001
LVDd, cm	4.80 (4.60, 5.10)	4.80 (4.50, 5.00)	4.85 (4.60, 5.20)	4.90 (4.60, 5.30)	<0.001
IVST, cm	0.90 (0.90, 1.00)	0.90 (0.80, 1.00)	0.90 (0.90, 1.00)	0.90 (0.90, 1.00)	0.118
LVPWT, cm	0.90 (0.80, 1.00)	0.90 (0.80, 1.00)	0.90 (0.90, 1.00)	0.90 (0.90, 1.00)	0.003
LVMI, g/m^2^	72.7 (63.0, 84.3)	70.9 (61.4, 81.6)	73.7 (64.5, 85.3)	79.1 (66.4, 91.6)	<0.001
LVEF, %	63.0 (60.0, 65.0)	63.0 (61.0, 66.0)	63.0 (60.0, 65.0)	63.0 (58.0, 66.0)	0.004
LVH, n (%)	105 (5.1)	36 (3.6)	54 (5.9)	15 (9.9)	<0.010
LVEF < 50%, n (%)	132 (6.4)	46 (4.6)	67 (7.3)	19 (12.5)	<0.001

Data are presented as median (interquartile range) or n (%).

ACEI/ARB = angiotensin-converting enzyme inhibitor/angiotensin receptor blocker, BMI = body mass index, CCB = calcium channel blocker, CHD = coronary heart disease, DBP = diastolic blood pressure, eGFR = estimated glomerular filtration rate, HDL-C = high-density lipoprotein cholesterol, IVST = interventricular septal thickness, LA = left atrium, LDL-C = low-density lipoprotein cholesterol, LVDd = left ventricular end-diastolic diameter, LVEF = left ventricular ejection fraction, LVH = left ventricular hypertrophy, LVMI = left ventricular mass index, LVPWT = left ventricular posterior wall thickness, NYHA = New York Heart Association, SBP = systolic blood pressure, WBC = white blood cell count.

### 3.2. Association between CONUT nutritional status and LVH

Univariable logistic regression analysis showed that, compared with participants with normal nutritional status, those with mild malnutrition had higher odds of LVH (odds ratio [OR] = 1.689, 95% confidence interval [CI]: 1.101–2.620, *P* = .018), while those with moderate/severe malnutrition had nearly threefold higher odds of LVH (OR = 2.938, 95% CI: 1.524–5.409, *P* < .001). After multivariable adjustment, these associations remained statistically significant. Mild malnutrition was associated with higher odds of LVH (OR = 1.597, 95% CI: 1.033–2.495, *P* = .037), and the association was stronger for moderate/severe malnutrition (OR = 2.810, 95% CI: 1.445–5.223, *P* = .002). In the final multivariable model, hypertension was also positively associated with LVH (OR = 1.980, 95% CI: 1.227–3.191, *P* = .005), whereas higher eGFR (OR = 0.980, 95% CI: 0.969–0.993, *P* = .002) and CCBs use (OR = 0.430, 95% CI: 0.260–0.711, *P* < .001) were inversely associated with LVH (Table 3).

**Table 3:** Univariable and multivariable logistic regression analyses for left ventricular hypertrophy (LVH).

Characteristic	Univariable			Multivariable		
	OR	95% CI	*P* value	OR	95% CI	*P* value
**CONUT nutritional status**						
Normal nutrition	Reference			Reference		
Mild malnutrition	1.689	1.101–2.620	.018	1.597	1.033–2.495	.037
Moderate/severe malnutrition	2.938	1.524–5.409	<.001	2.810	1.445–5.223	.002
Age, yr	1.018	0.999–1.038	.068	1.011	0.989–1.034	.325
BMI, kg/m^2^	0.959	0.911–1.008	.107			
Male (vs Female)	1.393	0.934–2.101	.108			
Hypertension (Yes vs No)	1.468	0.988–2.176	.056	1.980	1.227–3.191	.005
Diabetes (Yes vs No)	1.61	0.947–2.615	.065	1.596	0.918–2.662	.084
CHD (Yes vs No)	0.71	0.477–1.052	.089	0.702	0.445–1.100	.124
eGFR	0.979	0.969–0.990	<.001	0.980	0.969–0.993	.002
Glucose	1.036	0.958–1.107	.329			
Triglycerides	0.865	0.692–1.048	.172			
Statin use (Yes vs No)	0.788	0.529–1.169	.238			
ACEI/ARB use (Yes vs No)	1.184	0.796–1.755	.401			
β-blocker use (Yes vs No)	0.922	0.620–1.367	.687			
CCB use (Yes vs No)	0.624	0.417–0.928	.021	0.430	0.260–0.711	<.001
Diuretic use (Yes vs No)	1.402	0.903–2.133	.122			

ACEI/ARB = angiotensin-converting enzyme inhibitor/angiotensin receptor blocker, BMI = body mass index, CCB = calcium channel blocker, CHD = coronary heart disease, CI = confidence interval, CONUT = Controlling Nutritional Status, eGFR = estimated glomerular filtration rate, LVH = left ventricular hypertrophy, OR = odds ratio.

### 3.3. Clinical characteristics according to left ventricular systolic function

Among the 2066 participants, 132 (6.4%) had reduced left ventricular systolic function (LVEF < 50%). The distribution of CONUT nutritional status differed significantly between participants with preserved and reduced LVEF (*P* < .001). Compared with participants with LVEF ≥ 50%, those with LVEF < 50% had a lower proportion of normal nutritional status (34.8% vs 49.4%) and higher proportions of mild malnutrition (50.8% vs 43.7%) and moderate/severe malnutrition (14.4% vs 6.9%). Participants with LVEF < 50% were also older and more frequently male, and had higher prevalences of diabetes and CHD (all *P* < .05). Significant between-group differences were also observed in neutrophil count, glucose, TC, LDL-C, and albumin. Regarding echocardiographic parameters, participants with LVEF < 50% had significantly larger LA and LVDd and substantially higher LVMI than those with LVEF ≥ 50% (all *P* < .001) (Table 4).

**Table 4:** Baseline characteristics according to left ventricular systolic function.

Characteristic	Total	LVEF ≥ 50%	LVEF < 50%	*P* value
	(n = 2066)	(n = 1934)	(n = 132)	
**General characteristics**				
Age, yr	55.2 ± 9.9	55.0 ± 9.8	57.6 ± 10.4	.016
Male, n (%)	1121 (54.3)	1032 (53.4)	89 (67.4)	.002
BMI, kg/m^2^	26.1 (23.8, 29.0)	26.1 (23.8, 29.0)	26.8 (24.2, 29.6)	.117
Smoking, n (%)	866 (41.9)	813 (42.0)	53 (40.2)	.671
Drinking, n (%)	357 (17.3)	339 (17.5)	18 (13.6)	.252
SBP, mm Hg	139.1 ± 30.5	138.9 ± 30.2	142.1 ± 35.2	.396
DBP, mm Hg	84.6 ± 19.0	84.5 ± 19.0	85.3 ± 19.8	.689
Hypertension, n (%)	800 (38.7)	747 (38.6)	53 (40.2)	.728
Diabetes, n (%)	270 (13.1)	240 (12.4)	30 (22.7)	<.001
CHD, n (%)	1112 (53.8)	1028 (53.2)	84 (63.6)	.019
**Nutritional status**				
**CONUT nutritional status, n (%**)				<.001
Normal nutrition	1002 (48.5)	956 (49.4)	46 (34.8)	
Mild malnutrition	912 (44.1)	845 (43.7)	67 (50.8)	
Moderate/severe malnutrition	152 (7.4)	133 (6.9)	19 (14.4)	
**Laboratory findings**				
WBC, 10^9^/L	7.1 ± 4.4	7.1 ± 4.5	7.5 ± 2.6	.305
Neutrophil count, 10^9^/L	4.1 ± 2.7	4.1 ± 2.7	4.7 ± 2.4	.032
Lymphocyte count, 10^9^/L	2.1 (1.7, 2.5)	2.1 (1.7, 2.5)	2.0 (1.7, 2.5)	.806
Glucose, mmol/L	5.8 ± 2.4	5.8 ± 2.4	6.2 ± 2.6	.041
Triglycerides, mmol/L	1.8 ± 2.0	1.8 ± 1.9	1.8 ± 3.0	.924
Total cholesterol, mg/dL	153.9 (126.9, 182.0)	155.5 (128.4, 182.9)	137.5 (113.3, 168.2)	<.001
HDL-C, mmol/L	1.1 ± 0.8	1.1 ± 0.8	1.1 ± 0.8	.177
LDL-C, mmol/L	2.6 ± 0.9	2.6 ± 0.9	2.4 ± 0.9	.026
Albumin, g/L	40.2 (37.5, 42.8)	40.3 (37.6, 42.9)	38.4 (35.9, 41.4)	<.001
**Echocardiographic parameters**				
LA, cm	3.3 (3.1, 3.6)	3.3 (3.1, 3.6)	4.0 (3.5, 4.4)	<.001
LVDd, cm	4.8 (4.6, 5.1)	4.8 (4.6, 5.0)	6.0 (5.6, 6.5)	<.001
IVST, cm	0.9 (0.9, 1.0)	0.9 (0.9, 1.0)	0.9 (0.8, 1.0)	.167
LVPWT, cm	0.9 (0.8, 1.0)	0.9 (0.8, 1.0)	0.9 (0.8, 1.0)	.963
LVMI, g/m^2^	72.7 (63.0, 84.3)	71.7 (62.5, 82.2)	101.9 (84.6, 127.2)	<.001
LVEF, %	63.0 (60.0, 65.0)	63.0 (61.0, 66.0)	41.0 (37.0, 45.0)	<.001

BMI = body mass index, CHD = coronary heart disease, CONUT = Controlling Nutritional Status, DBP = diastolic blood pressure, GSP = glycated serum protein, HDL-C = high-density lipoprotein cholesterol, IVST = interventricular septal thickness, LA = left atrium, LDL-C = low-density lipoprotein cholesterol, LVDd = left ventricular end-diastolic diameter, LVEF = left ventricular ejection fraction, LVMI = left ventricular mass index, LVPWT = left ventricular posterior wall thickness, SBP = systolic blood pressure, WBC = white blood cell count.

### 3.4. Association between CONUT nutritional status and reduced LVEF

In univariable logistic regression analysis, compared with normal nutritional status, mild malnutrition was associated with higher odds of LVEF < 50% (OR = 1.648, 95% CI: 1.120–2.425, *P* = .011), while moderate/severe malnutrition was associated with nearly threefold higher odds (OR = 2.969, 95% CI: 1.688–5.221, *P* < .001). After multivariable adjustment, the association for mild malnutrition was no longer statistically significant (OR = 1.182, 95% CI: 0.779–1.803, *P* = .433), whereas moderate/severe malnutrition remained independently associated with reduced LVEF (OR = 2.350, 95% CI: 1.259–4.239, *P* = .006). In the final multivariable model, male sex (OR = 1.760, 95% CI: 1.183–2.652, *P* = .006), diabetes (OR = 1.789, 95% CI: 1.097–2.851, *P* = .017), ACEI/ARB use (OR = 1.879, 95% CI: 1.100–3.197, *P* = .020), and β-blocker use (OR = 2.654, 95% CI: 1.648–4.333, *P* < .001) were positively associated with reduced LVEF, whereas higher eGFR (OR = 0.974, 95% CI: 0.963–0.984, *P* < .001) and CCB use (OR = 0.151, 95% CI: 0.093–0.241, *P* < .001) were inversely associated with reduced LVEF (Table 5).

**Table 5:** Univariable and multivariable logistic regression analyses for reduced left ventricular ejection fraction (LVEF < 50%).

Characteristic	Univariable			Multivariable		
	OR	95% CI	*P* value	OR	95% CI	*P* value
**CONUT nutritional status**						
Normal nutrition	Reference			Reference		
Mild malnutrition	1.648	1.120–2.425	.011	1.182	0.779–1.803	.433
Moderate/severe malnutrition	2.969	1.688–5.221	<.001	2.35	1.259–4.239	.006
Age, yr	1.021	1.004–1.039	.018			
BMI, kg/m^2^	1.04	0.997–1.085	.066	1.039	0.992–1.087	.1
Male (vs Female)	1.809	1.244–2.632	.002	1.76	1.183–2.652	.006
CHD (Yes vs No)	1.542	1.070–2.223	.02			
Hypertension (Yes vs No)	1.066	0.744–1.528	.728			
Diabetes (Yes vs No)	2.076	1.352–3.188	.001	1.789	1.097–2.851	.017
eGFR	0.976	0.966–0.985	<.001	0.974	0.963–0.984	<.001
Glucose	1.062	1.001–1.127	.046			
Triglycerides	0.896	0.750–1.072	.23	0.83	0.677–0.993	.055
Statin use (Yes vs No)	1.667	1.160–2.397	.006			
ACEI/ARB use (Yes vs No)	1.978	1.384–2.826	<.001	1.879	1.100–3.197	.02
β-blocker use (Yes vs No)	2.157	1.491–3.119	<.001	2.654	1.648–4.333	<.001
CCB use (Yes vs No)	0.438	0.302–0.636	<.001	0.151	0.093–0.241	<.001
Diuretic use (Yes vs No)	1.241	0.837–1.840	.283	1.528	0.875–2.695	.139

ACEI/ARB = angiotensin-converting enzyme inhibitor/angiotensin receptor blocker, BMI = body mass index, CCB = calcium channel blocker, CHD = coronary heart disease, CI = confidence interval, CONUT = Controlling Nutritional Status, eGFR = estimated glomerular filtration rate, LVEF = left ventricular ejection fraction, OR = odds ratio.

## 4. Discussion

In this large retrospective cross-sectional study of adults, CONUT-defined nutritional status was significantly associated with left ventricular structural and functional abnormalities. Both mild and moderate/severe malnutrition remained significantly associated with higher odds of LVH, with a stronger association observed in the moderate/severe malnutrition group. Moderate/severe malnutrition was independently associated with reduced LVEF. These findings suggest that worsening nutritional status assessed by the CONUT score is associated with a greater burden of left‑ventricular structural abnormalities, and more advanced malnutrition may be associated with overt systolic dysfunction.

The CONUT score is an objective tool for assessing nutritional status that incorporates serum albumin, lymphocytes, and TC. It does not require anthropometric measurements or subjective evaluations. These indicators are easily accessible and exhibit strong generalizability. Albumin, an indicator of nutritional status in stable patients, has anti-inflammatory, antioxidant, and antithrombotic properties. Hypoalbuminemia has been repeatedly found to correlate with higher all-cause mortality across observational cohorts.^[21]^ States of elevated inflammation coincide with suppressed albumin synthesis, and reduced albumin concentrations often correlate with higher systemic inflammatory burden.^[22,23]^ TC is also regarded as an indicator of nutritional status.^[24]^ In a prior population-based analysis by Gnanenthiran et al lower TC concentrations among men were observed alongside higher prevalence of multimorbidity, frailty, and anemia, with frailty more common at lower cholesterol levels.^[25]^ Low TC levels are frequently observed alongside cachexia, malnutrition, malignant tumors and other chronic conditions that correlate with unfavorable clinical outcomes.^[26]^ Lymphocytes indicate immune function, where decreased levels suggest increased inflammation and a compromised nutritional status.^[27]^ The CONUT score evaluates a patient’s nutritional status by assessing protein reserves, immune function, energy expenditure, and inflammation, as all 3 markers are associated with malnutrition and inflammation.^[28]^

Prior studies on prognosis assessment and CONUT score have predominantly focused on cancer or liver conditions. Yoshida et al^[29]^ reported independent cross-sectional correlations between moderate-to-severe CONUT elevation and higher postoperative morbidity rates in patients with esophageal cancer. The research team concluded that CONUT score is an effective tool for assessing nutritional status before esophagectomy. A similar conclusion was reached for nasopharyngeal carcinoma patients.^[30]^ However, research focusing specifically on CVDs in this context is limited.

The link between nutritional status, especially malnutrition, and prognosis of patients with CVD has attracted growing research attention. A prior study indicated that malnutrition quantified by the CONUT score was correlated with higher all-cause mortality proportions in patients with acute myocardial infarction.^[31]^ Research has shown that elevated CONUT scores showed independent correlations with higher rates of cerebrovascular events within 1 year in older adults with non-ST-elevation myocardial infarction.^[32]^ A meta-analysis of CHD patients yielded comparable findings.^[33]^ Research indicates that CONUT score acts as a correlative marker for unfavorable clinical profiles in patients with both acute and chronic heart failure patients.^[34,35]^ Multiple existing meta-analyses have demonstrated that elevated CONUT scores are linked to worse survival outcomes in patients with heart failure, and poorer nutritional status correlates with higher mortality risk.^[36,37]^ One study indicated that both elevated CONUT scores and severe malnutrition showed independent cross-sectional correlations with higher long-term all-cause mortality. The 10-year all-cause mortality rate in patients with hypertension increased progressively with higher CONUT scores and worsening malnutrition levels.^[38]^ Hypertension is the most common cause of LVH. The prevalence of LVH varies from 20% in patients with mild hypertension to nearly 100% in those with severe or complicated hypertension.^[39]^ Large transmural myocardial infarctions are frequently accompanied by regional ventricular hypertrophy within non-infarcted segments, a pattern that coexists with postinfarction ventricular remodeling driven by volume overload.^[40]^

The present findings are consistent with accumulating evidence that poor nutritional status, as assessed by the CONUT score, frequently coexists with adverse cardiovascular profiles in adult populations, including individuals with hypertension and other cardiovascular conditions.^[41]^ This coexistence may partly reflect shared inflammatory and metabolic pathways linking malnutrition with cardiovascular abnormalities.^[9,42]^ Chronic systemic inflammation is common across a range of cardiovascular conditions, and previous studies have shown elevated levels of proinflammatory cytokines and acute-phase proteins, particularly in individuals with hypertension.^[43]^ Activated inflammatory pathways may increase catabolic demand and contribute to deterioration of nutritional status. Vitamin D deficiency is common in malnourished patients^[44]^ and is strongly associated with metabolic syndrome^[45]^ and CVDs such as hypertension.^[46]^ Vitamin D status correlates with blood pressure levels, possibly via effects on vascular endothelial and smooth muscle function as reported in prior research.^[47]^ A meta-analysis indicated that malnutrition quantified by CONUT correlates with higher proportions of all-cause death and major adverse cardiovascular events in patients with CHD.^[48]^ Albumin synthesis is influenced by nutritional intake and systemic inflammation.^[49]^ Serum albumin levels reflect both systemic inflammation and nutritional status. Inflammation is pivotal in both the onset and advancement of atherosclerosis as well as its acute clinical outcomes.^[50]^ Hypoalbuminemia may suggest ongoing arterial damage and the advancement of atherosclerosis and thrombosis.^[49]^ Disrupted cholesterol homeostasis, a feature of altered innate immune activity, coexists with aggravated inflammatory profiles that correlate with atherosclerotic progression.^[51,52]^ Systemic inflammation and malnutrition are frequently observed alongside reduced serum cholesterol concentrations.^[28,53,54]^ Patients with low cholesterol might not undergo aggressive statin treatment, potentially partially explaining the higher prevalence of adverse cardiac profiles observed among participants with elevated CONUT scores due to relatively low statin use. Malnutrition status often coincides with impaired immune function.^[55]^ A decrease in lymphocyte count is independently and significantly associated with negative clinical outcomes in CHD.^[56,57]^ A low total lymphocyte count is associated with increased mortality and serves as a correlative marker for unfavorable outcomes.^[58,59]^

We observed cross-sectional correlations between malnutrition and multiple cardiac parameters in this cohort. Hypoalbuminemia, low TC and reduced lymphocyte counts were concurrently present among participants with malnutrition and elevated LVMI. These nutritional and inflammatory markers were also correlated with indicators of myocardial fibrosis and ventricular stiffness, as well as impaired cardiac systolic and diastolic function. Such concurrent correlative patterns imply overlapping biological pathways that may coexist with both abnormal nutritional status and ventricular structural changes, though causal pathways cannot be confirmed from this cross-sectional dataset.^[60,61]^

Further clinical interpretation can be expanded by integrating the concept of the cold-and-dry heart failure phenotype proposed in prior clinical research.^[62]^ Classified via Stevenson hemodynamic stratification criteria, the cold-and-dry phenotype features peripheral hypoperfusion without evident pulmonary or systemic congestion, and this clinical profile is disproportionately observed among elderly hospitalized patients with inadequate nutritional intake, persistent low-grade inflammation and chronic volume depletion. The 3 laboratory indicators integrated into the CONUT score collectively reflect composite nutritional and inflammatory derangements that frequently coincide with this patient subgroup: hypoalbuminemia mirrors depleted protein reserves, reduced TC corresponds to sustained catabolic turnover, and lymphopenia aligns with persistent inflammatory activation. Consistent with observational evidence documented in the cited literature, chronic inflammatory load and long-term catabolic status coexist in individuals presenting both malnutrition and the cold-and-dry phenotype, and these concurrent metabolic-inflammatory disturbances correlate with markers of adverse ventricular remodeling, elevated LVMI and impaired systolic performance: findings consistent with the cross-sectional correlative trends identified in our cohort. From a clinical translational perspective, routine implementation of CONUT score screening may function as a simple auxiliary stratification metric to identify elderly inpatients prone to the cold-and-dry phenotype who exhibit concurrent higher prevalence of LVH, thereby broadening the clinical utility of nutritional evaluation for risk stratification among cardiovascular hospitalized populations.

## 5. Limitations

First, limitations related to study design and sampling: this is a single-center retrospective cross-sectional study, which may introduce selection bias. Critically, cross-sectional data cannot support any causal inference, and only statistical correlations between CONUT score and LVH-related indices can be reported rather than confirm directional causal effects. Long-term prospective cohort studies with serial nutritional and echocardiographic follow-up are needed to verify the correlative relationships observed herein. Second, the CONUT score only incorporates 3 laboratory indicators and may not fully reflect comprehensive nutritional status. Third, this work only reports population-level statistical correlations and lacks in-depth exploration of molecular mechanisms such as the mediating roles of inflammatory factors and oxidative stress; relevant basic experimental research is warranted to clarify potential biological pathways in the future.

## 6. Conclusion

In this retrospective cross-sectional study of adults, poorer CONUT-defined nutritional status was associated with a higher prevalence of left ventricular structural and functional abnormalities. Both mild and moderate/severe malnutrition were independently associated with higher odds of LVH, whereas only moderate/severe malnutrition remained independently associated with reduced LVEF after multivariable adjustment. These findings suggest that CONUT-defined nutritional status may provide supplementary information for cardiovascular risk stratification and the identification of individuals with cardiac structural or functional abnormalities.

## Acknowledgments

We thank all the participants for their commitment to this study.

## Author contributions

**Conceptualization:** Guan-Ying Yang, Jing Tang.

**Funding acquisition:** Guan-Ying Yang, Jing Tang, Yan-Hong Li.

**Investigation:** Guan-Ying Yang, Jing Tang.

**Methodology:** Guan-Ying Yang, Jing Tang.

**Supervision:** Jing Tang.

**Validation:** Jing Tang.

**Data curation:** Long Yang, Yan-Hong Li.

**Formal analysis:** Long Yang, Yan-Hong Li.

**Writing – original draft:** Guan-Ying Yang.
